# Vitamin D Deficiency in Children and Adolescents with Type 1 Diabetes

**DOI:** 10.4274/jcrpe.430

**Published:** 2011-12-06

**Authors:** Ajda Mutlu, Gül Yeşiltepe Mutlu, Elif Özsu, Filiz Mine Çizmecioğlu, Şükrü Hatun

**Affiliations:** 1 Kocaeli University, Medical Faculty, Department of Pediatrics, Kocaeli, Turkey; 2 Kocaeli University, Medical Faculty, Department of Pediatrics, Division of Pediatric Endocrinology and Diabetes, Kocaeli, Turkey; +90 262 303 72 28+90 262 303 70 03filizcizmeci@gmail.comKocaeli University, Medical Faculty, Department of Pediatrics, Division of Pediatric Endocrinology and Diabetes, Kocaeli, Turkey

**Keywords:** Vitamin D, type 1 diabetes, children and adolescents

## Abstract

**Objective:** To investigate the frequency and effects of vitamin D  deficiency in children with type 1 diabetes (T1D) in a region which is known to have a high rate of vitamin D deficiency among adolescents.

**Methods:** In this prospective cross-sectional study, 120 children and adolescents with T1D (55 girls and 65 boys) aged 3-20 years were  evaluated. Serum 25-hydroxyvitamin D [25(OH)D], parathormone (PTH), and alkaline phosphatase (ALP) levels were measured. Hemoglobin A1c levels and daily insulin requirement were also evaluated.  Classification of vitamin D status was made according to the American Academy of Pediatrics (AAP)/LWEPS’s recommendations. The patients were divided into 2 groups according to their vitamin D status and also according to the season of the year in which 25(OH)D sampling was done.

**Results:** Serum 25(OH)D levels revealed vitamin D deficiency or  insufficiency in 38% of the patients. Higher PTH levels were found in the patient group whose mean 25(OH)D level was <20 ng/mL as compared to the group whose mean 25(OH)D level was >20 ng/mL (p<0.05). Only 11% of patients had secondary hyperparathyroidism. The 25(OH)D levels of patients whose serum samples were taken in summer  and spring months were significantly different (p<0.05). There were no significant correlationsbetween 25(OH)D level and daily insulin dose.

**Conclusion:** Although we could not show a significant association between vitamin D deficiency and metabolic parameters, the frequency of vitamin D deficiency in T1D children is substantial. Vitamin D status should be assessed also in patients who do not have signs of rickets.

**Conflict of interest:**None declared.

## INTRODUCTION

There is a growing interest in the non-skeletal effects of vitamin D in recent years (1). There are some reports suggesting that vitamin D deficiency has a negative effect on insulin sensitivity and that it increases type 2 diabetes prevalence in adults (2,3,4,5). Some studies establishing a relationship between vitamin D and insulin sensitivity in children have also been published (6,7).  Recently, high rates of vitamin D deficiency have been reported in children with type 1 diabetes (T1D) and it is emphasized that this situation may have a negative effect on bone health (8,9,10,11,12,13). A positive effect of vitamin D administered in high doses (4000 IU/day) on metabolic control has also been reported (14). However, prevalence of vitamin D deficiency in children with T1D and its effects on metabolic control, insulin requirement and lipid levels have not yet been fully clarified. In this study, we aimed to investigate the frequency and effects of vitamin D deficiency in children with T1D. A high rate of vitamin D deficiency is encountered among adolescents in our region (15).

## MATERIALS AND METHODS

The study group consisted of one hundred twenty patients with T1D who were being followed in our pediatric endocrinology outpatient clinic. The mean age of patients was 12.7±3.8 years (range: 2.9-20.1 years). Fifty four percent of the patients (n=65) were boys and 46% (n=55) were girls.  None of the patients were taking a vitamin D supplement. Serum 25-hydroxyvitamin D [25(OH)D], parathormone (PTH), and alkaline phosphatase (ALP) levels were measured. Hemoglobin A1c (HbA1c) levels (mean of the measurements of the preceding year) and daily insulin requirement of the patients were evaluated. The season in which 25(OH)D samples were taken was also recorded. The participants were divided into two groups according to their pubertal stage. Group 1 consisted of prepubertal children (n=29) and group 2 - of children who had reached puberty (n=91). Because vitamin D status is associated with sunlight exposure, a variable which changes with the season of the year, the participants were categorized according to 25(OH)D sampling season: winter (December 22-March 21), spring (March 22-June 21), summer (June 22-September 21), and fall (September 22-December 21). Vitamin D status was classified according to the American Academy of Pediatrics (AAP)/LWEPS’s recommendations on cut-off levels for states of vitamin D. A 25(OH)D level of <5 ng/mL (<12.5 nmol/L) was considered as severe deficiency, a level between 5 and 15 ng/mL (12.5-37.5 nmol/L) as deficiency, a level of 15-20 ng/mL (37.5-50 nmol/L) as insufficiency, and a level of 20-100 ng/mL (50-250 nmol/L) as normal (sufficient) (16).  Our patients were also divided into 2 groups according to their vitamin D status. Those with 25(OH)D levels <20 ng/mL were grouped as vitamin D insufficient and deficient,  and those with 25(OH)D levels >20 ng/mL as normal with regard to vitamin D status. Serum 25(OH)D level was measured by ELISA reader and the microELISA method.  Serum level of ALP was measured using a Beckman CX-9 autoanalyzer. Serum PTH was measured by an original assay using Roche Diagnostics E-170 Modular Analytics immunoanalyzer equipment. The manufacturer’s normal range for PTH was 15-65 pg/mL and intra- and interassay CVs were 2.8 and 3.4%, respectively. Secondary hyperparathyroidism was defined as a PTH level greater than 65 pg/mL. HbA1c levels were measured by high-performance liquid chromatography (HPLC). The statistical analyses were performed with SPSS 13.0 software for Windows (SPSS Inc., Chicago, IL, USA). Mean values were compared using the Student’s t-test and Analysis of Variance (ANOVA). Pearson’s correlation analysis was used to reveal the relationships between 25(OH)D levels and daily insulin requirement of patients. The results were expressed as mean±SD. A p-value of less than 0.05 was considered statistically significant.

## RESULTS

The demographic and biochemical features of the patients are shown in Table 1. Serum 25(OH)D levels in 37.5% (n=45) of the patients revealed vitamin D deficiency or insufficiency. 0.8% (n=1) of patients had severe vitamin D deficiency. 21.7% (n=26) had vitamin D deficiency, and 15% (n=18) had vitamin D insufficiency. The 25(OH)D status level was normal (sufficient) in 60% (n=72) of the patients. A small fraction of the patients, 2.5% (n=3), had vitamin D excess. No significant differences were found between the 25(OH)D levels of girls and boys. The mean 25(OH)D level of the girls was 25.5±21.1 ng/mL (range: 4.6-101) and that of the boys was 25.8±10.7 ng/mL (range: 10.9-56.4), (p>0.05). Only five girls (9%) wore concealing clothes for religious reasons. The serum 25(OH)D levels of these five girls were 9, 13.7, 11.6, 101, and 16.1 ng/mL; the mean 25(OH)D level was 30.3±39.5 ng/mL (median= 13.7 ng/mL). Twenty four percent (n=29) of the study group was prepubertal and 76% (n=91) was pubertal. The mean 25(OH)D levels in the prepubertal and pubertal groups were similar (26.4±11.9 and 25.4±17.4 ng/mL, respectively, p>0.05). The characteristics of the prepubertal and pubertal participants are given in Table 2. There was no difference between prepubertal and pubertal patients in terms of metabolic control and vitamin D status. In the 45 patients (37.5%) whose mean 25(OH)D level was lower than 20 ng/mL, the mean PTH level was 44.6±24 pg/mL and the mean ALP level was 237±137.6 U/L. In the 75 patients (62.5%) whose mean 25(OH)D level was higher than 20 ng/mL, the mean PTH level was 35.5±12.8 pg/mL and the mean ALP level was 237±103.4 U/L. PTH levels in the group whose mean 25(OH)D level was lower than 20 ng/mL were significantly higher than in the other subgroup whose mean 25(OH)D level was greater than 20 ng/mL (p<0.05). Only 13 patients (10.8%) had secondary hyperparathyroidism. There was no significant difference in terms of daily insulin requirement and HbA1c% in the two groups (Table 3). Also, there was no significant correlation between 25(OH)D level and daily insulin dose (r=-0.07, p>0.05).  The rates of 25(OH)D samples taken at different time of the year were as follows: 42% in the summer, 23% in the winter, 21% in the spring, and 14% in the fall. The mean 25(OH)D levels of patients according to the seasons were respectively: 30.4±10.4 ng/mL, 24.8±26.7 ng/mL, 17.5±7.3 ng/mL and 25±11.2 ng/mL. One-way ANOVA showed significant differences between the means (p<0.05). The significant result was caused by the difference in 25(OH)D levels of patients whose serum samples were taken in the summer and those of patients whose samples were taken in the spring. Mean serum PTH, ALP and 25(OH)D levels of patients according to season are shown in Table 4.

**Table 1:**
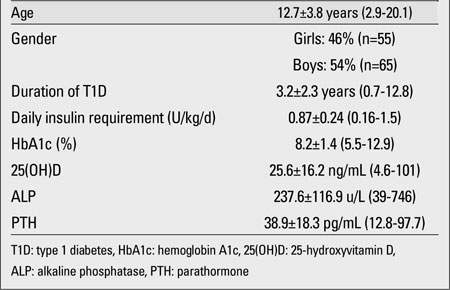
Demographic and biochemical features of patients

**Table 2:**
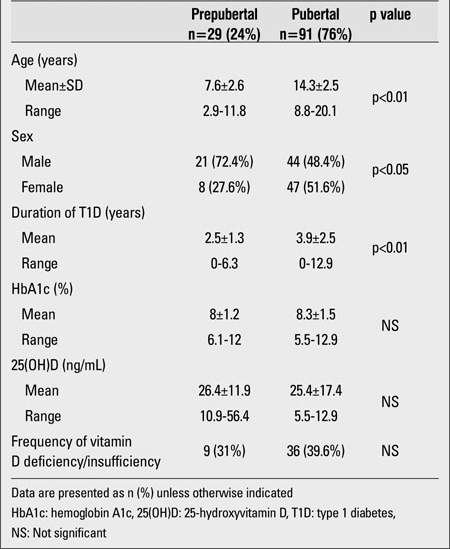
The comparison of prepubertal and pubertal participants

**Table 3:**
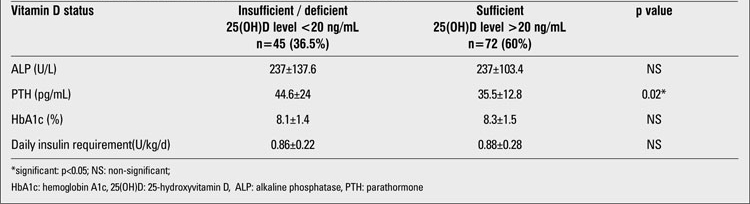
The comparison of vitamin D sufficient and insufficient/deficient patients

**Table 4:**
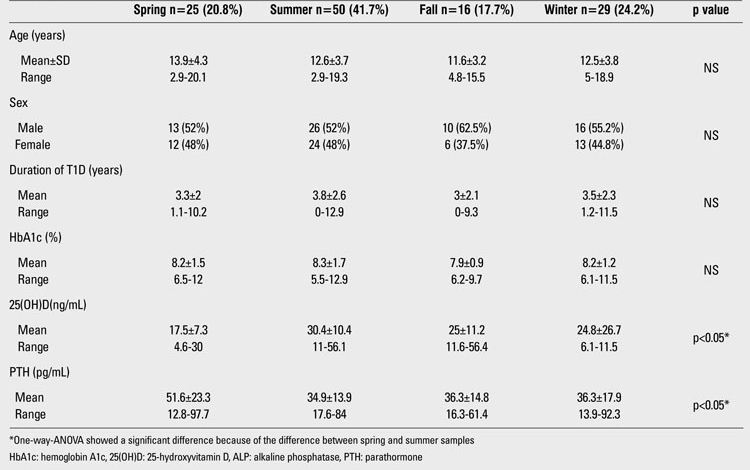
Mean serum 25(OH)D, ALP and PTH levels of patients according to season

## DISCUSSION

In recent years, studies suggesting that vitamin D deficiency correlates with the severity and frequency of T1D and that vitamin D supplementation reduces the risk of developing T1D have been reported  (17,18). On the other hand, although the number of studies on frequency of vitamin D deficiency in children and adolescents with T1D is limited, this frequency was found to be highly variable and may be as low as 15% or as high as 90.6% (10,11,13,19,20) (Table 5). Studies indicating that T1D patients have lower vitamin D levels than control groups have also been reported (8,9,10,12). In this study, we found the prevalence of vitamin D deficiency in children with T1D as 21.7% and our results were similar to those reported by Svoren et al (11) and Tunc et al (19). The reasons for the different frequencies given from different countries for vitamin D deficiency could be related to the variability of vitamin D deficiency definition (Table 5). Geographical environment and latitude may have influenced this variability (e.g., the latitudes of countries/ cities listed in Table 5 were: Boston: 42°19’ North, Switzerland: 47°00’ North, Qatar: 25°15’ North, Ankara 39°55’ and Kocaeli: 40°45’ North). Despite the high frequency of vitamin D deficiency (65%) in healthy adolescents in our region, we found the frequency of vitamin D deficiency in T1D children lower than that reported for healthy subjects (15). However, the limitation of this present study is the absence of an age- and gender-matched control group. Although the mean PTH level of the vitamin D insufficient group was below the cut-off value for hyperparathyroidism, it was significantly higher than that of the vitamin D sufficient group. We observed secondary hyperparathyroidism only in 10.8% of all patients whose vitamin D level was lower than 20 ng/mL. Janner et al (20) had found the prevalence of secondary hyperparathyroidism low in vitamin D deficient T1D patients as well. These findings indicate that all vitamin D deficient T1D patients do not have secondary hyperparathyroidism. Similarly, in a study from our region, secondary hyperparathyroidism frequency was very low (3%) among vitamin D deficient/insufficient adolescent girls who were known to be otherwise healthy (15). There are recent publications discussing the effects of vitamin D supplementation and even active vitamin D treatment on the metabolic control of diabetic patients (14,21). On the other hand, the number of studies on the relation of 25(OH)D levels with daily insulin requirement and metabolic control in children is limited. Johnson et al (22) found an inversely proportional correlation between fasting blood sugar level and 25(OH)D in pediatric outpatients. Svoren et al (11) and Tunc et al (19) showed that low serum 25(OH)D levels were associated with poor metabolic control in T1D patients. In our study, we did not find a significant correlation between serum 25(OH)D levels and HbA1c or duration of diabetes. Similarly, Janner et al (20) had reported that there was no relationship between HbA1c levels and vitamin D status in T1D children and adolescents. The daily insulin requirements of our subjects also did not change with their vitamin D status. In conclusion, although we could not show a significant association between vitamin D deficiency and metabolic parameters, the frequency of vitamin D deficiency in T1D children can be substantial. Vitamin D status of these children should be assessed in terms of bone health. Even in the absence of signs of rickets, vitamin D supplementation must be suggested to vitamin D deficient T1D children.

**Table 5:**
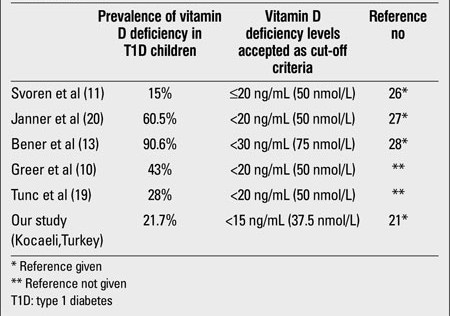
Recent studies investigating vitamin D deficiency prevalence in T1D children
